# The Fremantle Back Awareness Questionnaire: cross-cultural adaptation, reliability, and validity of the Italian version in people with chronic low back pain

**DOI:** 10.1186/s12891-024-07420-2

**Published:** 2024-04-11

**Authors:** Marco Monticone, Carolina Maurandi, Elisa Porcu, Federico Arippa, Benedict M. Wand, Giorgio Corona

**Affiliations:** 1 Department of Surgical Sciences, University of Cagliari, Cagliari, Italy; 2 Ambulatorio di quartiere, Cagliari, Italy; 3 Rehabilitation Medicine and Neurorehabilitation, P.O. San Martino, Oristano, Italy; 4 Department of Mechanical, Chemical, and Materials Engineering, University of Cagliari, Cagliari, Italy; 5 The Faculty of Medicine, Nursing & Midwifery and Health Sciences, The University of Notre Dame Australia, Fremantle, WA Australia; 6 Studio Fisioterapico Corona, Cagliari, Italy

**Keywords:** Low back pain, Rehabilitation, Rasch analysis, Validity, Reliability, Measurement error

## Abstract

**Background and aim:**

There is evidence to suggest that assessing back-specific altered self-perception may be useful when seeking to understand and manage low back pain (LBP). The Fremantle Back Awareness Questionnaire (FreBAQ) is a patient-reported measure of back-specific body perception that has never been adapted and psychometrically analysed in Italian. Hence, the objectives of this research were to cross-culturally adapt and validate the Italian version of this outcome measure (namely, the FreBAQ-I), to make it available for use with Italians suffering from chronic LBP.

**Methods:**

The FreBAQ-I was developed by forward and backward translation, review by a committee skilled in patient-reported measures and test of the pre-final version to assess its clarity, acceptability, and relevance. The statistical analyses examined: structural validity based on Rasch analysis; hypotheses testing by investigating correlations of the FreBAQ-I with the Roland Morris Disability Questionnaire (RMDQ), a pain intensity numerical rating scale (PI-NRS), the Pain Catastrophising Scale (PCS), and the Tampa Scale of Kinesiophobia (TSK) (Pearson’s correlations); reliability by internal consistency (Cronbach’s alpha) and test–retest repeatability (intraclass correlation coefficient, ICC (2,1)); and measurement error by determining the minimum detectable change (MDC). After the development of a consensus-based translation of the FreBAQ-I, the new outcome measure was delivered to 100 people with chronic LBP.

**Results:**

Rasch analysis confirmed the substantial unidimensionality and the structural validity of the FreBAQ-I. Hypothesis testing was considered good as at least 75% of the hypotheses were confirmed; correlations: RMDQ (*r* = 0.35), PI-NRS (*r* = 0.25), PCS (*r* = 0.41) and TSK (*r* = 0.38). Internal consistency was acceptable (alpha = 0.82) and test–retest repeatability was excellent (ICC (2,1) = 0.88, 95% CI: 0.83, 0.92). The MDC_95_ corresponded to 6.7 scale points.

**Conclusion:**

The FreBAQ-I was found to be a unidimensional, valid, and reliable outcome measure in Italians with chronic LBP. Its application is advised for clinical and research use within the Italian speaking community.

## Background

Low back pain (LBP) is a very common condition with a highly variable course[1]. Most episodes improve considerably within 6 weeks [2]; however, about two-thirds of persons still report some pain at 3 and 12 months, leading to chronic ill health[3, 4]. Chronic LBP has a wide range of deleterious effects on the individual, limiting functional capacity, work participation, and social engagement, as well as negatively impacting personal relationships, and mental and physical health [2].

An extensive research effort over many years has suggested multiple factors that might impact the chronic LBP experience, including changes in the way the back is perceived or experienced by the individual. Previous studies have reported that people with chronic LBP represent the back differently when asked to draw how it feels to them [5], have reduced tactile acuity [6], deficits in proprioception [7], reduced motor-imagery ability [8], and changes in tactile processing similar to the spatial neglect seen following cerebrovascular accidents [9].

Based on these premises, the Fremantle Back Awareness Questionnaire (FreBAQ) –a self-report questionnaire designed to assess back-specific body perception– was specifically developed for persons with chronic LBP [10]. The questionnaire was shown to be feasible, reliable, and valid, by means of associations with measures of pain duration, pain intensity, disability, and pain catastrophising [10]. Further, a later study on chronic LBP demonstrated the FreBAQ’s unidimensionality and acceptable internal consistency, as well as offering further support for the relationship between FreBAQ scores and clinical status, including measures of fear-avoidance and psychological distress [11].

The FreBAQ represents a helpful tool for assessing warning signs of compromised self-perception of the lower back in people with chronic LBP [10, 11] and in women with lumbopelvic pain during pregnancy and postpartum [12]. This questionnaire seems a promising instrument for identifying additional factors involved in the persistence of back problems, and could serve to guide targeted treatment strategies [10, 11, 13]. Indeed, preliminary studies suggest that treatment programs aimed at improving disturbed body perception (through sensorimotor retraining) may have positive effects on pain and function in individuals with non-specific LBP [14, 15].

However, the quality of a patient-reported outcome measure (PROM) may differ noticeably when cross-culturally adapted and used in a country different to where it was initially developed [16]. Well-established methodological criteria are recommended when validation studies are performed [17]. Application of these criteria are designed to ensure the quality of the new measurement tool and permit more confident comparison of findings across populations.

The FreBAQ has previously been adapted and psychometrically examined in Japanese, Dutch, German, Turkish, Chinese, Indian, Spanish and Persian populations [13, 18–24], but an Italian version (FreBAQ-I) has not yet been cross-culturally adapted and psychometrically analysed in a similar population. Therefore, the purpose of this research was to develop a FreBAQ-I and examine its psychometric properties in Italians suffering from chronic LBP.

## Methods

The COSMIN (COnsensus-based Standards for the selection of health status Measurement Instruments) guidelines were adopted [16].

The Institutional Review Board endorsed this cross-sectional study (no. 7/16, April 5th 2016). The research was carried out in accordance with the ethical and humane principles of research specified in the Declaration of Helsinki.

### Participants

This research engaged people attending an outpatient Hospital Rehabilitation Unit, meeting the following inclusion criteria: diagnosis of chronic non-specific LBP (i.e. a pain localised below the costal margin and above the inferior gluteal folds lasting for more than three months, without a distinguishable, specific, patho-anatomical cause or disease [25]); people speaking Italian as their first language (as well as those who had an adequate knowledge of Italian) and aged over 18. Exclusion criteria: acute (lasting up to one month) and subacute non-specific LBP (lasting up to three months); specific LBP (i.e. fracture, spinal deformity, disc herniation, canal stenosis, spondylolisthesis, or infections); peripheral or central neurological disorders assessed by means of imaging (e.g. radiographs, CT scans, or MRI) and/or anamnesis; systemic illness (including rheumatologic diseases); cognitive disorders (Mini Mental State Examination of < 24); recent myocardial infarctions; any past cerebrovascular accidents; and not capable or reluctant to give informed consent.

Participants were assessed by two physical and rehabilitation medicine physicians, who were under the supervision of the principal investigator (MM). Both physicians had at least a fifteen years’ experience, were involved in the assessments of the participants during the research process but not in the treatment procedure. Those who satisfied the criteria for inclusion were provided with information about the research aims and procedures and invited to sign a written informed consent form. After that, demographic and clinical characteristics were collected, and all participants completed the outcome measures listed below. Participants were invited to fill in the FreBAQ-I a second time, 7–10 days after their initial assessment to side-step variations in symptoms associated with possible memory effects [26].

### Cross-cultural adaptation

The Italian translation and adaptation of the FreBAQ was performed following the American Association of Orthopaedic Surgeon Outcomes Committee’s recommended protocol and according to the standards for good practice used in the translation and cultural adaptation procedure for PROM [27, 28].

#### Step 1: Italian translation

The original FreBAQ [10] was independently translated into Italian by two bilingual professionals with distinct backgrounds and some experience in the PROM field. They strove to select terms capturing the connotative meanings of the source text and –at the same time– reflecting everyday-spoken language. Conflicts between translations were examined by the principal investigator and then settled by consensus, in order to consolidate a preliminary Italian version.

#### Step 2: Back-translation Into English

Two native English-speaking bilingual professional translators, separately back-translated the preliminary adaptation. Then, the principal investigator and the translators checked and clarified potential inconsistencies between the different versions and agreed an advanced Italian version of the questionnaire.

#### Step 3: Expert Committee

This advanced version was submitted to a team of six bilingual clinicians, methodologists, and translators. They investigated the idiomatic, semantic, and theoretical similarity of items and response categories. This phase finished when a consensus was reached on a prefinal version.

#### Step 4: Test of the prefinal version

A pilot test was then performed to explore intelligibility, appropriateness, cultural relevance, and potential ambiguity of the prefinal version. Cognitive interviews were performed by a qualified psychologist after administering the tool to 10 persons with chronic LBP, representatives of the target population. The team of experts examined the findings of this test in order to detect any useful refinement and then agreed the final version of FreBAQ-I. These deliberations are available from the corresponding author on request.

### Acceptability and feasibility

**Fig. 1:**
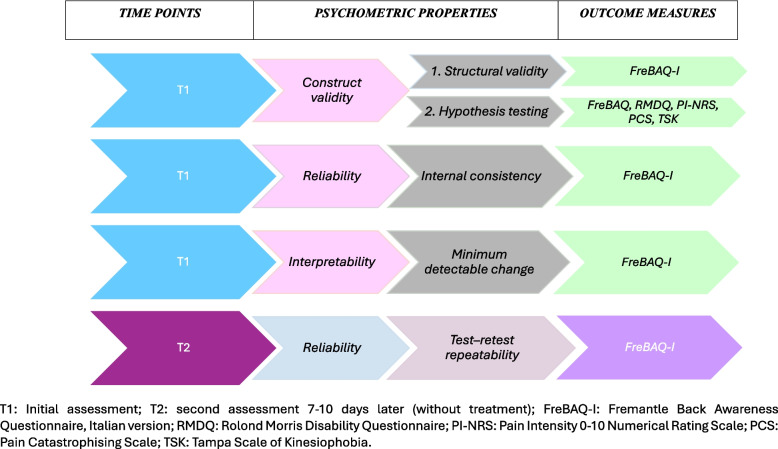
The study procedure

Participants were interviewed about the comprehensibility of each part of the questionnaire, and the data were verified for missing or multiple answers. The time to compile each questionnaire was gathered (Fig. 1).

### Psychometric properties

#### Construct validity

It represents the degree to which the scores of a measurement instrument are consistent with hypotheses, with regard to internal relationships, or relationships with scores of other instruments or differences between relevant groups [29] and was assessed by structural validity and hypothesis testing.

*1. Structural validity* (i.e. the degree to which the scores of a measurement instrument are an adequate reflection of the dimensionality of the construct to be measured [29])*.* Rasch analysis (Winsteps software v. 4.8.0) examined the FreBAQ-I using the rating scale model (because all items shared the same rating scale structure). Our detailed iterative procedure has been reported in previous studies [30, 31]. In short, the following psychometric issues were investigated:

a) diagnostic assessment of the rating categories, by investigating whether each response category was being used consistently and effectively; for that, the transition thresholds between categories (i.e. the points where two adjacent categories have an equal probability to be endorsed) and average category measures should be ordered from less to more on the underlying latent continuum [32];

b) internal construct validity, assessed checking how well the observed responses to the items align with the responses predicted by the Rasch model, using chi-square fit statistics (infit and outfit mean-square statistics, MnSq). Based on the sample size, values from 0.75 to 1.30 [33] were considered as indicating an acceptable fit;

c) reliability, in terms of both person reliability index and item reliability index [32], providing an estimate of the degree of replicability (across different samples) of person and item placements along the trait continuum (range 0–1; coefficients > 0.80 are considered as good, > 0.90 as excellent). High reliability levels (of persons or items) mean that there is a high probability that persons (or items) estimated with high Rasch measures actually do have higher measures than persons (or items) estimated with low measures [33].

d) unidimensionality of the scale, examining the unexplained variance after the Rasch dimension is extracted, as obtained by a Principal Component Analysis of the residuals (PCAr). Additional factors are not likely to be present in the residuals if the eigenvalue of the first residual component is < 2 [33];

e) local item dependence. For any pair of items, no residual correlation > 0.20 (above the average observed residual correlation) should be detected once the variable under measurement (Rasch factor) has been filtered out [34].

*2. Hypothesis testing*, which takes place when hypotheses are formulated a priori on the relationships of scores on the instrument under investigation with scores deriving from other measures evaluating related or dissimilar constructs, by also describing the expected direction (i.e. positive or negative) and magnitude (i.e. low, moderate, large) [29]*.* Based on what previously assumed in a previous study on the same matter [11], it was hypothesized a priori the FreBAQ-I would achieve positive moderate correlations (from 0.30 to 0.60) with measures of disability, and catastrophizing, and low correlations (< 0.30) with measures of pain intensity and kinesiophobia. Pearson’s correlation coefficients were calculated, and construct validity was considered as satisfactory if at least 75% of the hypotheses was reached [26].

#### Reliability

It represents the degree to which the measurement is free from measurement error [29] and was calculated as detailed below.

- 1. Internal consistency is the degree of interrelatedness among the items [29]) and was evaluated by calculating Cronbach’s alpha (values of > 0.70 being considered acceptable).
- 2. Test–retest repeatability is the degree to which the measurement is free from measurement error over time [29]) and was examined 7–10 days later without treatment using the intraclass correlation coefficient, ICC (2,1) (values of 0.70–0.85 were considered good and > 0.85 excellent) [26].
- 3. The standard error of measurement (SEM) is the difference between an amount that can be measured and its true value [29]) and was assessed using the formula:
  $$SEM=SD\sqrt{1-{ICC}_{\mathrm{2,1}}}$$

where SD represents the standard deviation of the measurements at baseline.

#### Interpretability

It represents the degree to which one can assign qualitative meaning to an instrument’s quantitative scores or change in scores [29] and was calculated by the minimum detectable change (MDC) (i.e. the change beyond measurement error [29]) by using the following equation:

$$MDC=SEM* z value*\sqrt{2}$$

A z value of 1.96 was used to derive a 95% confidence level MDC (MDC_95_).

Descriptive statistics were further calculated to identify floor/ceiling effects, which were present if > 15% of the scores achieve their lowest or highest potential value, respectively [26].

### Questionnaires

#### FreBAQ-I

This questionnaire quantifies distorted perception of the back. It is self-administered and includes 9 items. Each item is scored by a five-point response scale (range: 0 = ‘never’ up to 4 = ‘always’); the final score is obtained by summing the responses from each of the items and ranges from 0 to 36, with higher scores corresponding to greater levels of back-perception distortion [10].

#### Roland Morris Disability Questionnaire

This questionnaire assesses LBP related disability. It comprises 24 items, with a total score ranging from 0 (no disability) to 24 (highest level of disability) [35].

#### Pain Numerical Rating Scale (PI-NRS)

An 11-point pain numerical rating scale ranging from 0 (no pain at all) to 10 (the worst imaginable pain) was used [36], asking participants to rate their current pain intensity.

####  Pain Catastrophising Scale (PCS)

This is a 13-item self-administered questionnaire. People are asked to classify the frequency with which they experience the thoughts listed in the tool, based on a five-point scale, which ranges from 0 (never) to 4 (always). The total score is obtained summing up the scores of the individual items and can vary from 0 to 52 [37]. Higher scores denote greater levels of pain catastrophising.

####  Tampa Scale of Kinesiophobia (TSK)

This questionnaire is self-administered and composed of 13 items [38]. Each item is scored using a four-point Likert scale ranging from 1 (strongly disagree) to 4 (strongly agree), and the total score is calculated by adding the scores of the individual items (range 13–52). Higher values correspond to greater fear of movement [38].

All outcome measures were administered in their validated Italian versions [35, 37–41]. The FreBAQ-I was systematically distributed first, then the RMDQ, the NRS, the TSK and the PCS during the first assessment, respectively; only the FreBAQ-I was delivered during the second assessment. Statistical analyses were performed with STATA 13.1 software (StataCorp LP, College Station, TX, USA).

The sample size of 100 was determined to provide adequate statistical power for test–retest reliability, expecting to obtain, with 90% probability, an ICC of about 0.85, with the lower limit of the 95% CI not less than 0.75 [42]. Moreover, a sample size of 100 participants is able to ensure stability in Rasch item calibrations within ± 0.5 logits with 95% confidence [43].

## Results

### Participants

**Fig. 2:**
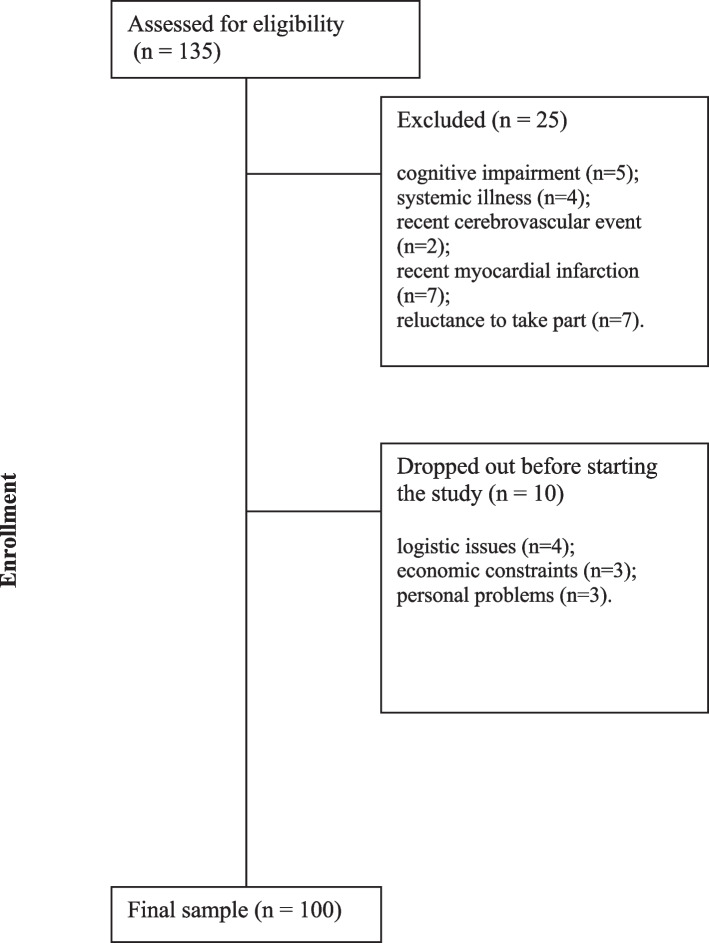
Flow chart of participants inclusion

One hundred and thirty-five persons with chronic non-specific LBP were consecutively assessed, of whom 25 were excluded. The reasons for exclusion were: cognitive impairment (*n* = 5); systemic illness (*n* = 4); recent cerebrovascular event (*n* = 2); recent myocardial infarction (*n* = 7); and reluctance to take part (*n* = 7). Of the remaining people, 10 dropped out before starting the study because of: logistic issues (*n* = 4); economic constraints (*n* = 3); or personal problems (*n* = 3). Hence, our final sample was comprised of 100 subjects. All participants returned to the hospital for a second assessment within a period of 7 to 10 days, facilitated by a telephonic follow-up conducted by a research assistant. Average pain duration was 49 months (SD 80). Socio-demographic and clinical characteristics of the participants are described in Tables 1 and 2 (Fig. 2).

**Table 1:** Socio-demographic characteristics of the study population (*n* = 100)

Age (years), mean ± SD		52.96 ± 15.63
Gender	Male	37
	Female	63
Married	Yes	63
	No	37
Employment	Students	3
	Employed	48
	Self-employed	12
	Domestic works	12
	Retired	23
	Unemployed	2
Education level	Primary school	3
	Middle school	24
	High school	36
	University	37
Smokers	Yes	18
	No	82
Body Mass Index (kg/m2), mean ± SD		24.47 ± 3.97

*SD* Standard deviation

**Table 2:** Scores (mean and standard deviation, SD) of the examined questionnaires: Fremantle Back Awareness Questionnaire (FreBAQ), Roland Morris Disability Questionnaire (RMDQ), Pain Intensity Numerical Rating Scale (PI-NRS), Tampa Scale of Kinesiophobia (TSK) and Pain Catastrophising Scale (PCS). Minimum–maximum possible scores for each tool are given between brackets

Measures (range)	Mean (SD)
FreBAQ (0–36)	9.68 (6.98)
RMDQ (0–24)	8.83 (4.98)
PI-NRS (0–10)	5.24 (1.73)
TSK (13–52)	32.59 (7.01)
PCS (0–52)	21.45 (11.17)

### Translation and cross-cultural adaptation

The adaptation process took four weeks to settle upon a culturally appropriate version. All the terms were easily forward and back translated and there was no need of any major local adjustment. The term “occasionally” of one rating category was translated in Italian with “qualche volta” because it was judged as more suitable for a middle response category, while the translation “occasionalmente” would have been hardly discernible in Italian from the adjacent category “rarely/raramente”.

The appropriateness of the whole procedure and related results was endorsed by the team of experts, who also reviewed the findings from the cognitive interviews and made only minor changes based on concerns raised by some participants, to enhance the questionnaire’s comprehensibility. After that, the principal investigator and experts confirmed the final version of the FreBAQ-I, in agreement with the developer (BMW).

### Acceptability

The questionnaire took 1.97 ± 1.13 min to complete. The questions were well received. No missing responses or multiple responses were observed, nor were any comprehension difficulties raised during the instrument completion.

### Scale psychometric properties

#### Construct validity

##### Structural validity

The 5-level rating scale of the FreBAQ-I showed a monotonical advance of both the transition thresholds between categories and the average category measure. According to the mean-square goodness-of-fit statistics, all items fitted the Rasch model (Table 3).

**Table 3:** Item calibrations (measure with standard error, SE), and fit statistics (infit and outfit mean-square statistics, MnSq) for the FreBAQ-I

Item	Measure (SE)	Fit (MnSq)	
		Infit	Outfit
1. My back feels as though it is not part of the rest of my body	0.68 (0.14)	0.86	0.80
2. I need to focus all my attention on my back to make it move the way I want it to	-0.34 (0.11)	1.06	1.17
3. I feel as if my back sometimes moves involuntarily, without my control	0.43 (0.13)	0.86	0.80
4. When performing everyday tasks, I don’t know how much my back is moving	0.17 (0.12)	0.88	0.86
5. When performing everyday tasks, I am not sure exactly what position my back is in	-0.55 (0.10)	1.03	1.05
6. I can’t perceive the exact outline of my back	-0.15 (0.11)	0.87	0.97
7. My back feels like it is enlarged (swollen)	0.28 (0.12)	1.27	1.00
8. My back feels like it has shrunk	0.72 (0.14)	1.16	0.91
9. My back feels lopsided (asymmetrical)	-1.24 (0.11)	1.23	1.23

The mean person ability was -0.97 logits (ability range from -3.06 to 3.34). The item reliability was 0.96, while person reliability was 0.75. The PCAr showed that FreBAQ was essentially unidimensional: the measured variable explained 50.3% of the variance in the data, while the secondary component explained only 9.9% of the variance (corresponding to an eigenvalue of 1.8). No local item dependence was detected, (i.e., no strong > 0.20 residual correlation between items was found).

##### Hypothesis testing

It was considered good as 3 out of 4 a priori hypotheses were confirmed (i.e., ≥ 75%). Related results are shown in Table 4.

**Table 4:** Hypothesis testing. Pearson’s correlation (r) between the Fremantle Back Awareness Questionnaire, Italian version (FreBAQ-I) and the other clinical measures (for all, *p* < 0.01)

	FreBAQ-I
Roland Morris Disability Questionnaire	0.35
Pain Intensity Numerical Rating Scale	0.25
Tampa Scale of Kinesiophobia	0.38
Pain Catastrophising Scale	0.41

#### Reliability

Cronbach’s α was 0.82. Test–retest reliability was found to be high: ICC (2,1) = 0.88 (95% CI: 0.83—0.92). The SEM was 2.44.

#### Interpretability

The MDC_95_ was 6.7 points.

The mean score for the FreBAQ-I was 9.68 points (SD 6.98). No ceiling or floor effects were detected in any of the used scales, including the FreBAQ-I.

## Discussion

This research describes the cross-cultural adaptation of the FreBAQ and the assessment of its validity, reliability, and measurement error, in Italian-speaking people with chronic non-specific LBP. The FreBAQ-I demonstrated unidimensionality, good validity, and adequate reliability. International recommendations were followed in the current study and all the steps suggested that the process of translation and cross-cultural adaptation was accurate and efficient. Our methodological approach comprised forward and backward translation, minor amendments by a team of experts, cognitive debriefing, and discussion and resolution through consensus among the committee members. The procedure established the initial conceptual, semantic, and content equivalence between source and target language. The final version was well accepted, and easily understood and self-administered. The respondent burden was minimal as the questionnaire needs only a few minutes to complete. Overall, the FreBAQ-I appears to be appropriate for everyday clinical practice.

### Construct validity

#### Structural validity

Our results corroborated the findings of the previous structural validations of this outcome measure [11, 13, 44]. The tool proved to be unidimensional (a key measurement requirement), with items acceptably fitting the mathematical model, and a rating scale functioning as expected. No local item dependence was found. In our sample the average difficulty of the items (endorsability) was about 1 logit lower than the mean sample ability (agreeability): in that condition the scale better assesses persons with moderate to high levels of disturbed body perception. These findings indicate that the item selection was appropriate and able to correctly measure the variable of interest. A minor deviation from unidimensionality was found in a sample of Indian people with chronic LBP but no issue was expected as for the clinical application of the FreBAQ in this population [22].

#### Hypothesis testing

The correlation with disability (RMDQ) was as expected (Table 3), indicating that higher levels of back pain related disability are associated with enhanced levels of body perception disruption relating specifically to the back. This is in line with the findings of the developers, where a moderate correlation with disability was observed (0.32)[11]. As predicted, we also noted a positive correlation between pain intensity and disrupted perception of the back, though this relationship was weaker than that noted for disability, also consistent with previous research [11]. Taken together, these results suggest that disrupted body perception is more strongly related to disability than pain in people with chronic LBP [11]. This issue was also seen in most other adapted studies of the FreBAQ (i.e. Japanese, Dutch, German, Turkish and Persian) [13, 18–20, 24], probably indicating disrupted body perception impacts more on the functional consequences of pain, rather than the experience of pain itself. A higher correlation with disability than pain was otherwise found in the Spanish sample (0.48 vs 0.38) [23]. Very low correlations with disability and pain were found in the Indian study [22].

With respect to catastrophizing, we noted a similar correlation to what was reported in the original English version (0.36) [11]. Our results are also consistent with results obtained from the Japanese (0.38) [13], Turkish (0.41) [20], Spanish (0.46) [23] and Persian (0.60) [24] cross-cultural adaptation studies. These findings support the idea of a relationship between high levels of pain catastrophizing and disrupted body perception. For kinesiophobia, our estimates are slightly higher than those reported by the original developers (0.26) [11], however this study utilized a different measure of kinesiophobia (i.e. the Fear Avoidance Beliefs Questionnaire, physical activity subscale), which makes direct comparison difficult. Our results are somewhat in line with the Turkish and Spanish estimates (0.60 and 0.37) [20, 23], while divergent from what was found in Japanese, Dutch and Persian samples (0.22, 0.10, and 0.17 respectively) [13, 18, 24], and, therefore, more analyses are recommended before firm conclusions can be drawn about the relationship between kinesiophobia and altered body perception.

### Reliability

The internal consistency of the FreBAQ-I was good (0.82) and quite similar to that reported by the developers (0.80) [11]. Similar results were found in other versions of the questionnaires: Japanese 0.80 [13]; Dutch 0.82 [18]; German 0.91 [19]; Turkish 0.87 [20]; Chinese 0.83 [21], Indian 0.91 [22], Spanish 0.82 [23] and Persian 0.74 [24].

Test–retest repeatability demonstrated an excellent level of agreement between the results on days 1 and 8 (ICC (2,1) = 0.88), a value higher than those reported in the original study (0.65) [10], while in the other validations, values were of 0.69 [18], 0.78 [23] and 0.90 [21]. The better reliability noted in this investigation may reflect the fact that no treatment was provided to participants between the two testing occasions, a control not enacted in the original study [10]. The measurement error of the FreBAQ-I was acceptable. Due to the high repeatability of the test–retest the SEM and MDC were rather low.

### Interpretability

The MDC_95_ demonstrated that a change of more than 7 points after a given intervention (~ 19% of the FreBAQ score range of 36 points) would not be the result of an error in measurement. Slightly different findings were achieved by the Dutch study (where the MDC was estimated at 10.8 points) [18] and the Persian study (2.52 points) [24], while the validation of the Chinese and the Spanish version of the FreBAQ reported an MDC_95_ of 5.99 and 5.12 points, respectively [21].

This study should acknowledge some limitations. First, the study design is cross-sectional and thus responsiveness and minimal important change could not be assessed. Second, no external anchor such as a global rating of change was used to assess clinical stability during the assessment of reliability and participants may have improved or worsened between the first and second assessment of the FreBAQ-I. Third, the association between back-related perceptual dysfunction and physical performance measures was not investigated as only questionnaires were employed. Fourth, relationships with other psychological characteristics (e.g. Pain Self-Efficacy Questionnaire or the Coping Strategies Questionnaire-27 revised) [45–47], or quality of life (e.g. the Short-Form Health Survey 36-items) [48], as well as with clinical tests that might have the ability to detect alterations in the sensorimotor system [49], were not examined. Fifth, our research was limited to people with chronic non-specific LBP and it is doubtful if these results can be expanded to individuals with other causes of lumbar pain (e.g. canal stenosis, fracture, or disk herniation) or pain of another duration. Therefore, studies in these populations are advised.

## Conclusions

The FreBAQ-I displays a one-factor structure, it is valid and reliable, and has an adequate measurement error. This Italian version can be recommended for use in clinical and research settings for the assessment of Italian-speaking people with chronic LBP.

## Data Availability

The datasets used and/or analysed during the current study are available from the first author under a reasonable request.

## Abbreviations

CI: Confidence Interval
CT: Computed Tomography
FreBAQ: Fremantle Back Awareness Questionnaire
FreBAQ-I: Fremantle Back Awareness Questionnaire-Italian
ICC: Intraclass correlation coefficient
ISPOR: International Society for Pharmacoeconomics and Outcomes Research
LBP: Low back pain
MDC: Minimum detectable change
MnSq: Mean-square statistics
MRI: Magnetic Resonance Imaging
PCAr: Principal Component Analysis of the residuals
PCS: Pain Catastrophising Scale
PI-NRS: Pain intensity numerical rating scale
PROM: Patient-reported outcomes measures
SD: Standard error
SEM: Standard error of measurement
TSK: Tampa Scale of Kinesiophobia
